# Hyperfractionated Radiotherapy with Concurrent Cisplatin/5-Fluorouracil for Locoregional Advanced Head and Neck Cancer: Analysis of 105 Consecutive Patients

**DOI:** 10.1155/2012/754191

**Published:** 2012-06-21

**Authors:** David Zaboli, Marietta Tan, Hrishikesh Gogineni, Spencer Lake, Katherine Fan, Marianna L. Zahurak, Barbara Messing, Karen Ulmer, Eva S. Zinreich, Marshall A. Levine, Mei Tang, Sara I. Pai, Ray G. Blanco, John R. Saunders, Simon R. Best, Joseph A. Califano, Patrick K. Ha

**Affiliations:** ^1^Johns Hopkins University School of Medicine, Baltimore, MD 21205, USA; ^2^Department of Otolaryngology-Head and Neck Surgery, Johns Hopkins Medical Institutions, 1550 Orleans Street, Room 5M06, David H Koch Cancer Research Building, Baltimore, MD 21231, USA; ^3^Case Western Reserve University School of Medicine, Cleveland, OH 44106, USA; ^4^Division of Oncology Biostatistics, Department of Oncology, Johns Hopkins Medical Institutions, Baltimore, MD 21231, USA; ^5^The Milton J. Dance, Jr. Head and Neck Center, Greater Baltimore Medical Center, Baltimore, MD 21204, USA; ^6^Department of Radiation Oncology, Sandra & Malcolm Berman Cancer Institute, Greater Baltimore Medical Center, Baltimore, MD 21204, USA; ^7^Department of Medical Oncology, Sandra & Malcolm Berman Cancer Institute, Greater Baltimore Medical Center, Baltimore, MD 21204, USA

## Abstract

*Objective*. We reviewed a cohort of patients with previously untreated locoregional advanced head and neck squamous cell carcinoma (HNSCC) who received a uniform chemoradiotherapy regimen. *Methods*. Retrospective review was performed of 105 patients with stage III or IV HNSCC treated at Greater Baltimore Medical Center from 2000 to 2007. Radiation included 125 cGy twice daily for a total 70 Gy to the primary site. Chemotherapy consisted of cisplatin (12 mg/m^2^/h) daily for five days and 5-fluorouracil (600 mg/m^2^/20 h) daily for five days, given with weeks one and six of radiation. All but seven patients with N2 or greater disease received planned neck dissection after chemoradiotherapy. Primary outcomes were overall survival (OS), locoregional control (LRC), and disease-free survival (DFS). *Results*. Median followup of surviving patients was 57.6 months. Five-year OS was 60%, LRC was 68%, and DFS was 56%. Predictors of increased mortality included age ≥55, female gender, hypopharyngeal primary, and T3/T4 stage. Twelve patients developed locoregional recurrences, and 16 patients developed distant metastases. Eighteen second primary malignancies were diagnosed in 17 patients. *Conclusions*. The CRT regimen resulted in favorable outcomes. However, locoregional and distant recurrences cause significant mortality and highlight the need for more effective therapies to prevent and manage these events.

## 1. Introduction

An estimated 630,000 people worldwide were diagnosed with head and neck squamous cell carcinoma (HNSCC) in 2008, representing 6% of all malignancies and making it the 6th most common cancer [1, 2]. In the United States, approximately 48,000 cases occurred in 2009, with 11,300 deaths [3]. The 5-year survival of all stages is approximately 60% [4]. However, two-thirds of patients are diagnosed at a locoregional advanced stage (III-IVb), with 5-year survival rates of 30 to 60% for these patients [5]. In the past decade, treatment of locoregional advanced HNSCC has shifted from primary surgery to organ preservation with combination chemoradiotherapy (CRT). The current approach attempts to achieve both organ preservation and function with outcomes superior to radiotherapy alone or surgery with postoperative radiotherapy [6–12]. A recent meta-analysis by Pignon et al. showed an absolute survival benefit of 6.5% at five years when chemotherapy was administered concomitantly with radiotherapy [13]. 

The mechanism for the survival benefit of CRT is thought to occur via increased tumor sensitization to the cytotoxic effects of radiotherapy, while providing adjuvant treatment for potential distant metastatic disease [7]. However, CRT has acute and long-term toxicities that can both limit treatment and increase morbidity. Furthermore, although several CRT regimens produce high rates of complete response at the primary site and regional neck nodes, there exists a high rate of failure to systemically eradicate micrometastases. This is demonstrated by the later occurrence of distant metastases, which account for many cancer-specific deaths. Therefore, despite the successful use of numerous CRT regimens and the development of multidisciplinary management, the prognosis of locoregional advanced HNSCC remains poor [14].

In this study, we review the outcomes of a heterogeneous cohort of patients with locoregional advanced HNSCC. All patients were treated with curative intent with a uniform CRT regimen that consisted of hyperfractionated radiotherapy and concurrent cisplatin/5-fluorouracil (5-FU). Outcomes evaluated were overall survival (OS), locoregional control (LRC), and disease-free survival (DFS).

## 2. Methods

### 2.1. Study Subjects

In an IRB-approved study design (Greater Baltimore Medical Center IRB number 07-044-11), the medical records of 105 patients with previously untreated, locoregional advanced, nonmetastatic stage III-IVB squamous cell carcinoma of the oropharynx, hypopharynx, or larynx were reviewed. All patients were treated at the Greater Baltimore Medical Center between 2000 and 2007. Patients with cancers of the salivary glands, sinuses, or unknown primary sites were excluded, as were patients with recurrent tumors or previous chemotherapy or radiation to the head or neck.

Patients were evaluated by a head and neck surgeon, medical oncologist, radiation oncologist, dentist, speech pathologist, nurse, and social worker. All cases were discussed prior to therapy and on a regular basis thereafter at multidisciplinary conference. All patients underwent comprehensive head and neck exam, including laryngoscopy and triple endoscopy when necessary, and imaging assessment with chest X-ray, CT, PET/CT, or MRI of the head and neck when appropriate. All patients had a histologic diagnosis of squamous cell carcinoma and were staged according to American Joint Committee on Cancer (AJCC) guidelines [15]. When possible, tumor samples were tested for HPV-16 DNA via in situ hybridization-catalyzed signal amplification as previously described [16]. Feeding gastrostomy tubes were routinely placed prior to initiation of CRT.

Patient demographic and clinical characteristics collected at baseline included age, gender, race, patient-reported weight loss, and Karnofsky Performance Status (KPS). In addition, history of tobacco and alcohol use was collected and categorized as follows: tobacco as nonsmoker, <20 pack-years (PY), 20–40 PY, 40–60 PY, or >60 PY; alcohol as nondrinker, <7 drinks/week (social), 7–14 drinks/week (moderate), or >14 drinks/week (heavy).

### 2.2. Chemoradiotherapy

Chemotherapy consisted of cisplatin (12 mg/m^2^) administered over one hour and 5-FU (600 mg/m^2^) over twenty hours, dosed for five consecutive days in an inpatient setting during weeks one and six of radiation therapy. Radiation therapy consisted of hyperfractionated doses of 125 cGy delivered twice daily for 28–33 days for a total dose of 70–75 Gy to the primary tumor site, 60 Gy to involved lymph nodes, and 50 Gy to uninvolved cervical and supraclavicular lymph nodes. Patients were treated with 6 MV photon beams, opposed lateral fields with focus blocks, and neck nodes were boosted with electron beam after a cumulative dose of 40 Gy. Treatment interruptions were minimized as much as possible, and a planned treatment break of one week was included after a cumulative dose of 40 Gy.

### 2.3. Assessment of Toxicity

Mucositis severity, scored according to the World Health Organization (WHO) oral toxicity scale, was collected from records of patient visits to speech language pathology and clinical notes of the radiation and medical oncologists. Duration of gastrostomy tube dependence was calculated from the initiation of CRT until the date of removal.

### 2.4. Neck Dissection

All but seven patients with clinical N2 or N3 disease at the time of presentation underwent planned neck dissection. Neck dissections were performed eight to twelve weeks after the completion of CRT. Determination of selective versus comprehensive neck dissection was made on an individual patient basis.

### 2.5. Patient Followup

After treatment, patients returned for followup every 2-3 months in years 1-2, every 3–6 months in years 3–5, and every 6–12 months thereafter, or sooner in the event of a clinical concern requiring closer scrutiny.

### 2.6. Statistical Analysis

The primary statistical endpoints of this study were OS, LRC, and DFS. Event time distributions were estimated with the method of Kaplan and Meier and compared using the log-rank statistic or the proportional hazards regression model. The simultaneous effect of two or more factors was studied using the multivariate proportional hazards model, which was reported with 95% confidence intervals (CIs). OS was calculated from the date of treatment initiation to the day of death or last followup. DFS was calculated from the date of treatment initiation to the day of recurrence or death, whichever came earlier. LRC was similarly calculated from the date of treatment initiation to the day of local or regional recurrence, whichever came first. Factors evaluated for prognostic value included age, gender, race, tumor site, histologic grade, AJCC stage, tumor stage, nodal stage, self-reported weight loss, KPS, pretreatment hemoglobin, tobacco and alcohol history, and HPV status. In proportional hazards regression models, variables were entered as categorical effects and the hazard ratios for these factors reflect either its presence or absence. All *P* values are two-sided. Computations were performed using the Statistical Analysis System.

## 3. Results

One-hundred five patients were included in this study. Their clinical characteristics are listed in Table 1. The median followup of surviving patients was 57.6 months (range 4.6 to 118.8). One patient died from pulmonary embolism during the first week of treatment. All other patients completed the regimen as described.

### 3.1. Overall Survival and Analysis of Patient Deaths

**Figure 1:**
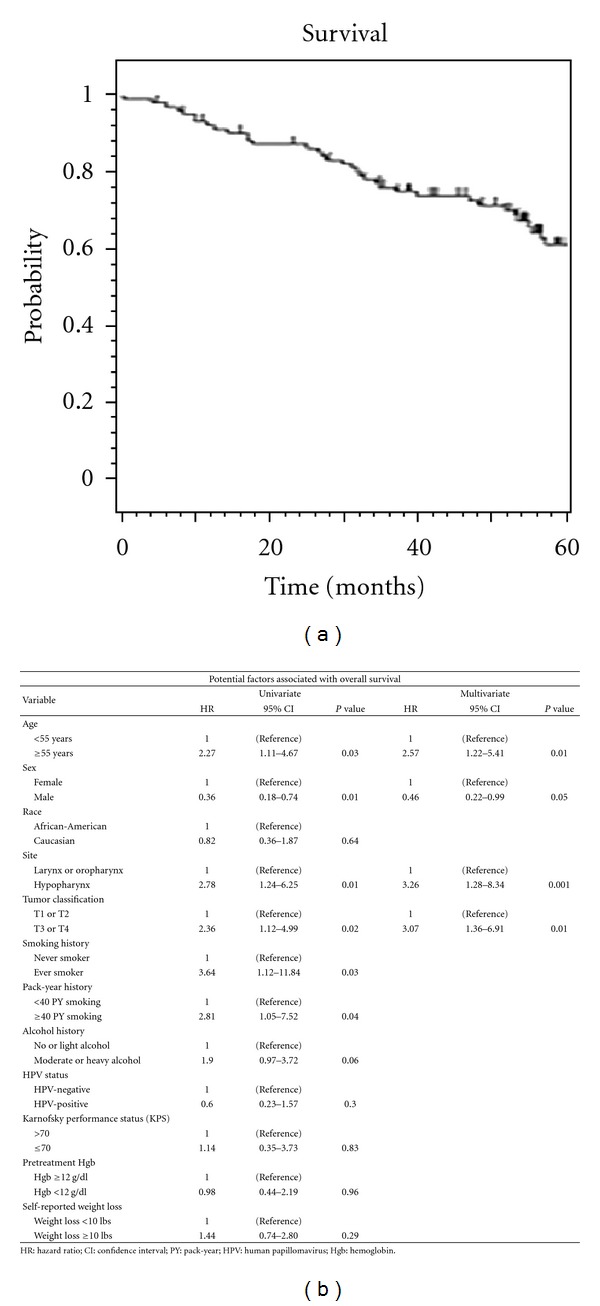
(a) Kaplan-Meier depiction of overall survival for all patients, (b) univariate and multivariate analyses of clinical characteristics potentially associated with overall survival.

As of July 2010, 66 (63%) patients were alive.  Figure 1(a) depicts overall survival for all patients. Median survival was 99 months, with 3- and 5-year overall survival rates of 77 and 63% for stage III and 72 and 58% for stage IV. Including both stages III and IV, the 3- and 5-year OS was 75 and 60%. Uni- and multivariate analyses of factors associated with survival are listed in Figure 1(b). On uni- and multivariate analysis, age greater than 55, primary hypopharyngeal cancers, and advanced T3/T4 tumors were associated with decreased survival, while male gender was associated with increased survival. On univariate analysis only, any smoking history and greater than 40 pack-year smoking history were associated with decreased survival. HPV-positive cancers of the oropharynx were associated with slightly decreased mortality; however, this was not statistically significant (*P* = 0.3). The distribution of deaths from primary head and neck cancer and other causes is shown in Table 2.

### 3.2. Locoregional Recurrence and Distant Metastases

**Figure 2:**
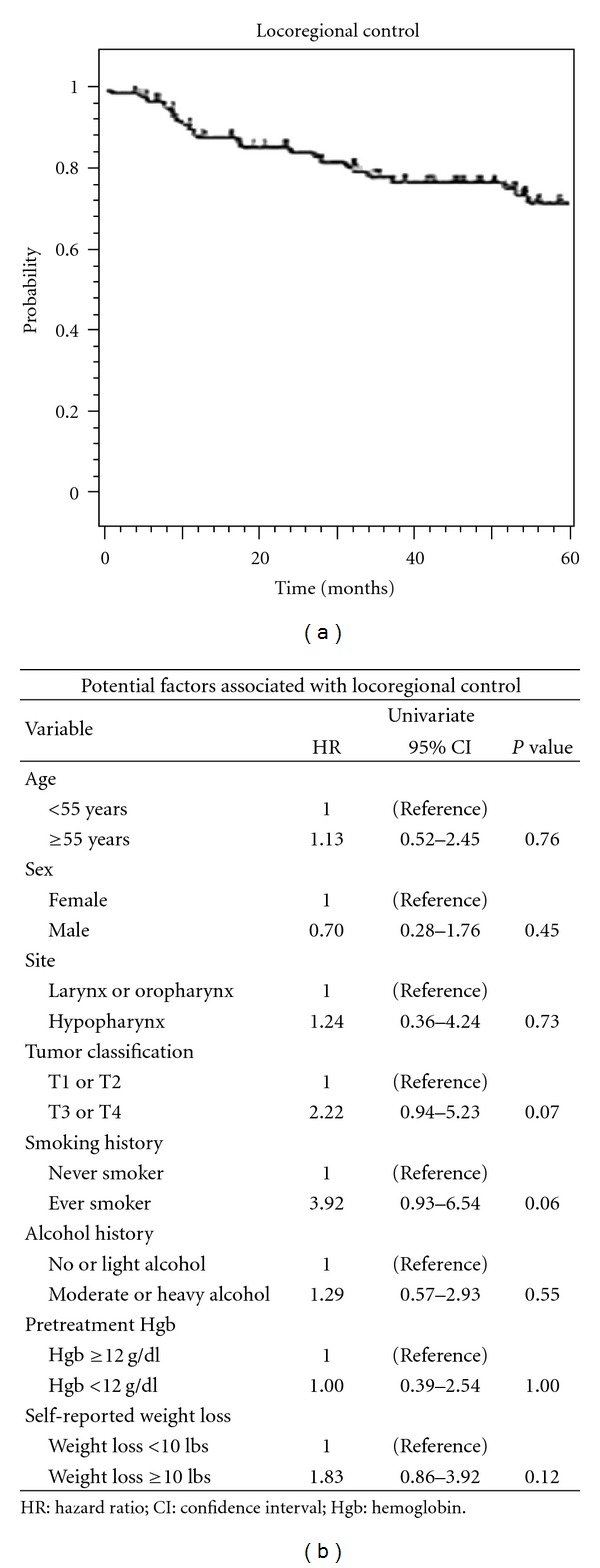
(a) Kaplan-Meier depiction of locoregional control for all patients, (b) univariate analysis of clinical characteristics potentially associated with locoregional control.

Locoregional recurrence occurred in 12 patients. The average time to locoregional recurrence was 13.8 months (range 6.3 to 33.9) after initiation of CRT. The 3- and 5-year rates of LRC were 76 and 68%, respectively (Figure 2(a)). The average survival after diagnosis of locoregional recurrence was 2.7 years. Univariate analysis of factors associated with LRC is listed in Figure 2(b).

Of the five patients who developed regional recurrences, three had undergone planned neck dissection following completion of CRT. Two of these three patients had viable carcinoma identified in the ipsilateral cervical lymph nodes, while the other patient had no evidence of viable tumor identified in any lymph nodes.

Distant metastases occurred in 16 patients (15%). The most common site of distant metastatic disease was the lungs (*n* = 12). The average time to diagnosis of distant metastasis was 14.1 months (range 3.2 to 31.9) after initiation of CRT. The average survival from time of diagnosis of distant metastasis was 1.3 years.

### 3.3. Disease-Free Survival

**Figure 3:**
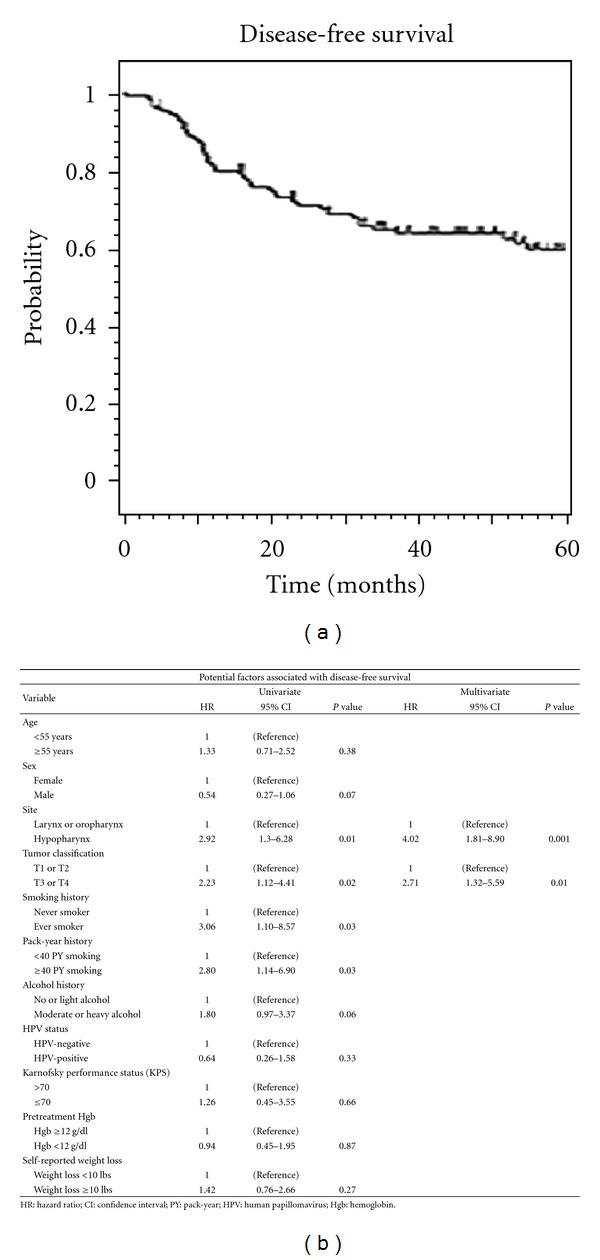
(a) Kaplan-Meier depiction of disease-free survival for all patients, (b) univariate and multivariate analyses of clinical characteristics potentially associated with disease-free survival.

 The average time to development of any recurrence or metastasis was 14.1 months (range 3.2 to 33.9) after initiation of CRT. Median disease-free survival was 96 months, with 3- and 5-year rates of 63 and 56%, respectively (Figure 3(a)). Uni- and multivariate analyses of factors associated with DFS are listed in Figure 3(b). Hypopharyngeal cancer and T3/T4 tumors were both significantly associated with decreased disease-free survival.

### 3.4. Second Primary Malignancies

Eighteen second primary malignancies (SPMs) were diagnosed in 17 patients after the completion of treatment. The most common SPM was lung (*n* = 7), followed by colorectal (*n* = 3), HNSCC (*n* = 2), prostate (*n* = 2), medullary thyroid carcinoma (*n* = 1), chronic lymphocytic leukemia (*n* = 1), multiple myeloma (*n* = 1), and cutaneous squamous cell carcinoma (*n* = 1). The average time to diagnosis of SPM was 33 months (range 3.0 to 63.2).

### 3.5. Toxicity

Data for mucositis grading was available for 66 patients (63%). Twenty-four patients (36%) had grade 3 mucositis, and 39 patients (59%) had grade 4 mucositis. Osteoradionecrosis was diagnosed in 5 patients (5%). Data on PEG use was available for 94/105 patients. The median duration of PEG use was 134 days (range 31–1570), which included patients who died with a PEG tube in place. Thirty-two of 94 patients (34%) required a PEG for greater than 6 months, and 10/94 patients (11%) required a PEG for greater than 12 months. Data on stricture development was available for 67 of 78 patients with oropharyngeal cancer. Thirteen out of these 67 patients (19%) developed a stricture, as reported previously [17].

### 3.6. Neck Dissection

Seventy-one of 105 patients were nodal stage N2 or N3, and 64 patients underwent neck dissection (61%). Of the seven patients with N2 or N3 disease who did not undergo neck dissection, two died prior to surgery, one was too ill to undergo surgery, one refused surgery, and reasons were not available for the other three patients. Residual carcinoma was identified in 18 patients (28%). This rate of viable carcinoma detected post-CRT is comparable to that in the cohort of patients with oropharyngeal cancer previously reported by Hillel et al. and is similar to other studies [18–20].

## 4. Discussion

The use of combination chemotherapy and radiation therapy as primary treatment for locoregional advanced HNSCC in medically fit patients has been well established as a treatment option in numerous trials and meta-analyses. Furthermore, altered fractionation radiotherapy with concomitant chemotherapy is a well-established treatment for locoregional advanced, nonmetastatic disease. The landmark study by Brizel et al. showed that hyperfractionated radiotherapy with cisplatin and 5-FU resulted in improved survival compared to radiation therapy alone [6]. Several subsequent clinical trials have reported outcomes based on different treatment regimens. The optimal regimen that maximizes efficacy and minimizes toxicity remains controversial. Therefore, we sought to review the efficacy of a uniform treatment regimen in a heterogeneous cohort with locoregional advanced HNSCC.

### 4.1. Efficacy of CRT Regimen

The results of the concurrent administration of CRT for 105 consecutive patients revealed favorable outcomes. Rates of overall survival and locoregional control were similar to those reported elsewhere. For example, two other studies reported 5-year overall survival rates of 59% and 40%, respectively, in cohorts of patients with stage III or IV disease who received similar radiation and the same chemotherapeutic agents [21, 22]. In another series of stage IV patients who received concurrent CRT, the 5-year OS was 46% [10]. Likewise, in the study by Brizel et al., which employed similar hyperfractionated radiotherapy and concurrent cisplatin and 5-fluorouracil in the same doses as our regimen, the 3-year OS was 55% and the LRC was 70% [6].

In our cohort, 12 patients (11%) developed locoregional recurrence, and 16 patients (15%) developed distant metastasis. This is comparable to other studies with similar CRT regimens, which have reported locoregional recurrence in 22–31% of patients and distant metastasis in 13–20% of patients [21, 22]. The patterns of failure were approximately equal between locoregional (*n* = 12) and distant sites (*n* = 16), which contrasts with several studies in which the majority of failures occur either at the primary tumor site or cervical lymph nodes [6, 12, 23]. Brockstein et al. reported the results of their experience with two main treatment regimens: Type 1, intensive induction chemotherapy followed by split-course chemotherapy, or Type 2, intensive, split-course hyperfractionated chemotherapy [5]. They reported a 5-year LRC rate of 31 and 17% for types 1 and 2, respectively, and a 5-year distant failure rate of 13 and 22% for types 1 and 2. Interestingly, the patients who received induction CRT were more likely to have locoregional control but were also more likely to have distant metastatic relapse. This has prompted some to suggest the role of induction chemotherapy in treating micrometastatic disease, as other trials have shown a reduction in rates of distant metastasis with the use of induction CRT [24].

### 4.2. Decreased Survival in Cancers of the Hypopharynx

In our cohort, there were 14 patients who presented with a primary cancer of the hypopharynx and one patient with cancers of the hypopharynx and oropharynx. Of the hypopharyngeal cancers, 13/14 (93%) were stage IV at presentation. Eleven of the 14 patients (79%) with hypopharyngeal cancer underwent neck dissection, and 6/11 (55%) had viable carcinoma (versus 28% of the overall cohort who underwent neck dissection). Eight of 14 patients (57%) had a recurrence, compared to 25/105 (24%) in the overall cohort. Furthermore, of the eight recurrences in the hypopharynx group, seven of them presented with distant metastases. These data reflect the poor overall survival of those with hypopharynx cancers in this cohort, which is similar to other published studies [25–28].

Patients with hypopharyngeal squamous cell carcinoma may therefore need a different therapeutic approach. Given the ongoing debate regarding the possibility of induction chemotherapy to better treat micrometastases, this method of CRT should be considered in a direct trial against concomitant CRT or perhaps a return to primary surgery with postoperative radiotherapy is warranted.

### 4.3. Toxicity

The high rate of Grade 3 or 4 mucositis observed in this cohort was consistent with other reports [29, 30]. With regard to the elective placement of PEG tubes, this is supported by the need to maintain or minimize the weight loss associated with CRT, which is between 10 to 14% [12, 30, 31]. In one study, elective versus nonelective use of PEG was compared, and elective PEG resulted in less weight loss and decreased length of hospitalization [32]. The median duration of PEG use and the rate of patients that required a PEG tube for longer than 6 or 12 months is similar to other studies [33–35]. Furthermore, the exact duration reported may be overestimated due to the retrospective nature of this study and the inability to determine exactly when the patients no longer used the PEG tube.

### 4.4. Second Primary Malignancies

Patients with head and neck cancer have an increased rate of second primary malignancies, estimated at 3–9% per year [2, 36]. These cancers affect the entire aerodigestive tract, are often related to smoking history, and are therefore of great significance in survivors of head and neck cancer [37]. Consistent with these studies, the high rate of second primary lung cancer detected in our patient cohort was not unexpected and was ultimately responsible for a significant fraction of patient deaths after they were without evidence of disease of head and neck cancer. In our cohort, 2 of 18 SPM were cancers of the head and neck, similar to another cohort of patients treated with CRT [37]. The average time to occurrence of SPM was 33 months (2.75 years) in our cohort, similar to other publications of 2.8 years [30, 37].

 One strength of this study included the large cohort of patients. In addition, treatment compliance was high, and few patients experienced unplanned treatment breaks or delays due to excessive toxicity. Furthermore, long-term followup was available. Only two patients were lost to followup immediately after treatment, and all but six remaining patients had records available from a provider at our institution within the previous year. This was also reflected in the median followup of surviving patients of 57.6 months. Other strengths included the utilization of a uniform CRT regimen, and a thorough chart and database review and availability of the medical records of several departments. Despite these strengths, one limitation to our study was its retrospective nature. Although efforts were made to collect toxicity data, this was often unavailable and not systematically recorded in patient notes; therefore, documentation of toxicity may have been underestimated and therefore may not be representative.

## 5. Conclusions

The results of this study show that the use of hyperfractionated radiotherapy with concurrent cisplatin and 5-fluorouracil remains an excellent regimen for the primary treatment of locoregional advanced head and neck cancer. However, the prognosis for patients who experience relapse remains poor, and research focused on predicting which patients will experience these events and what treatment modifications, if any, should be made in order to improve the outcomes for these patients is warranted. Lastly, the poor outcomes of patients with cancer of the hypopharynx merit consideration of ongoing research and novel therapies to lessen the mortality of this disease.

## Figures and Tables

**Table 1:** Patient characteristics at baseline (*n* = 105).

		Number of patients (%)
Age	Age in years, mean (range)	58.5 (40.2–78.5)
	<55 years	42 (40)
	≥55 years	3 (60)

Sex	Female	21 (20)
	Male	84 (80)

Race	Caucasian	90 (86)
	African-American	15 (14)

Site	Oropharynx^a^	78 (74)
	Hypopharynx^b^	15 (14)
	Larynx	13 (12)

Stage	III	30 (29)
	IV	75 (71)

Tumor classification (T)	T1	6 (6)
	T2	36 (34)
	T3	45 (43)
	T4	18 (17)

Nodal status (N)	N0	14 (13)
	N1	24 (23)
	N2	56 (53)
	N3	11 (10)

Histologic grade^c^	Well	5 (5)
	Moderately	44 (42)
	Poorly	45 (43)
	Unknown	11 (10)

Smoking history	No	23 (22)
	Yes	82 (78)
	<20 PY	21 (20)
	20–40 PY	21 (20)
	40–60 PY	18 (17)
	>60 PY	18 (17)
	Amount unknown	4 (4)

Alcohol history	No	8 (8)
	Yes	90 (86)
	Social	26 (25)
	Moderate	29 (28)
	Heavy	35 (33)
	Unknown	7 (7)

HPV status^d^	Positive	25 (34)
	Negative	20 (27)
	Unavailable	32 (43)

Karnofsky Performance Status	≤70	9 (9)
	80	29 (28)
	90	31 (30)
	100	27 (26)
	Unknown	9 (9)

Pretreatment Hgb	<12 g/dl	26 (25)
	≥12 g/dl	71 (68)
	Unavailable	8 (8)
Self-reported weight loss	None or less than 10 lbs	28 (27)
	Greater than 10 lbs	67 (64)
	Unknown	10 (10)

PY: pack-year; HPV: human papillomavirus; Hgb: hemoglobin.

^a^One patient presented with 2 primary cancers of the oropharynx.

^b^One patient presented with synchronous primary cancer of the oropharynx and hypopharynx.

^c^If the histologic grade was midway between well to moderately or moderately to poorly differentiated, the higher grade was chosen.

^d^For tumors of oropharyngeal primary site only.

**Table 2:** Causes of death (*n* = 39).

	Number of patients
Head and neck cancer	21
Comorbidity	6
Second primary malignancy	7
Unknown	5
